# An Improved Artificial Bee Colony Algorithm Based on Balance-Evolution Strategy for Unmanned Combat Aerial Vehicle Path Planning

**DOI:** 10.1155/2014/232704

**Published:** 2014-03-20

**Authors:** Bai Li, Li-gang Gong, Wen-lun Yang

**Affiliations:** ^1^School of Control Science and Engineering, Zhejiang University, Hangzhou 310027, China; ^2^School of Automation Science and Electrical Engineering, Beihang University, Beijing 100191, China

## Abstract

Unmanned combat aerial vehicles (UCAVs) have been of great interest to military organizations throughout the world due to their outstanding capabilities to operate in dangerous or hazardous environments. UCAV path planning aims to obtain an optimal flight route with the threats and constraints in the combat field well considered. In this work, a novel artificial bee colony (ABC) algorithm improved by a balance-evolution strategy (BES) is applied in this optimization scheme. In this new algorithm, convergence information during the iteration is fully utilized to manipulate the exploration/exploitation accuracy and to pursue a balance between local exploitation and global exploration capabilities. Simulation results confirm that BE-ABC algorithm is more competent for the UCAV path planning scheme than the conventional ABC algorithm and two other state-of-the-art modified ABC algorithms.

## 1. Introduction

Developments in automated and unmanned flight technologies have become an irresistible trend in many countries. As a matter of fact, unmanned combat aerial vehicles (UCAVs) have been of great importance to many military organizations throughout the world due to their capabilities to work in remote and hazardous environments [1]. Path planning is a critical aspect of the autonomous control module in UCAV, which aims to provide an optimal path from the starting point to the desired destination with the artificial threats and some natural constraints considered.

For the UCAV path planning scheme, an optimal solution corresponds to one path that minimizes the flight route, average altitude, fuel consumption, exposure to radar or artillery, and so forth [2]. With the development of the ground defending weapons, the difficulty of describing these artificial threats significantly becomes larger. Therefore, in order to deal with the increasing complexity when modeling a combat field, researchers have gradually shifted their interests away from deterministic algorithms [3–5].

Like most real-world optimization problems, finding the global optimum is enormously difficult. To avoid enumerating for the global optimums, evolutionary algorithms (EAs) have been well investigated and developed as a primary branch of the heuristic algorithms, such as genetic algorithm (GA) [6], differential evolution algorithm (DE) [7], ant colony optimization algorithm (ACO) [8], particle swarm optimization algorithm (PSO) [9], and artificial bee colony algorithm (ABC) [10]. Although these intelligent algorithms do not necessarily guarantee global convergence, some satisfying results can be acquired after all. That is why studies have been brought on developing new algorithms or modifying the existing ones in recent years [11]. For this UCAV path planning scheme, algorithms such as chaos theory based ABC (C-ABC) [12], immune GA (I-GA) [13], PSO [14], quantum-behaved PSO (Q-PSO) [15], master-slave parallel vector-evaluated GA (MPV-GA) [16], and intelligent water drops algorithm (IWD) [17] have been applied.

ABC algorithm is a swarm intelligence algorithm motivated by the foraging behaviors of bee swarms. In this algorithm, the bee swarm mainly consists of three components: the employed bees, the onlooker bees, and the scout bees [18]. Employed bees start the global exploration, then the qualified employed bees will be capable of attracting the onlooker bees to follow. At this point, following means exploiting locally around an employed bee. The qualification of each employed bee is determined by the roulette selection strategy. In the long run of the iterations, those unqualified employed bees eventually perish and scout bees will take their places.

Applications of ABC algorithm span the areas of image processing [19], structure identification [20], bioinformatics [21], neural network training [22], and so forth. At the same time, it is believed that ABC algorithm works well in the global exploration but poorly in the local exploitation [23]. Generally speaking, two prevailing ways have been taken to improve the conventional ABC algorithm. In the first way, strategies, for instance, Rosenbrock's rotational direction strategy [24], quantum theory [25], chaos theory [26], and Boltzmann selection strategy [27] from the outside world are introduced. The second way mainly focuses on combining ABC algorithm with some other intelligent algorithms. DE-ABC [28], PSO-ABC [29], and QEA-ABC [30] are typical examples. Apart from the two ways mentioned above, efforts have also been made on revising the crossover and mutation equations in the conventional ABC algorithm [21, 31, 32]. Especially, an improved ABC algorithm named I-ABC has shown fast convergence speed and accurate convergence precision in comparison with ABC algorithm and is regarded as a state-of-the-art version of ABC [32].

Viewing improvements ever made for ABC algorithm, attentions have seldom been paid to fully utilizing the convergence messages hiding in the iteration system [33, 34]. In other words, apart from the roulette selection strategy, the convergence status of a previous cycle may be regarded as feedback information to guide a subsequent cycle. In addition, it is noted that the generation of scout bees mainly intends for the escapement of premature convergence but scout bees are confirmed to be noneffective in some numerical cases [35]. Therefore, new rules may be needed to guide the scout bees so as to perform more efficiently. Moreover, it is believed that the exploration and the exploitation procedures need to match with each other so as to cooperate efficiently. That is, it is essential for an intelligent algorithm to capture a balance between global exploration and local exploitation.

In this paper, a novel ABC algorithm modified by a balance-evolution strategy is applied for this path planning scheme. In this new algorithm (which is named BE-ABC), convergence status in the iteration will be fully utilized so as to manipulate the exploration/exploitation accuracy and to make a tradeoff between local exploitation and global exploration. Besides, the rule guiding the scout bees is modified according to an overall degradation procedure. This work intends for some intensive research to evaluate the performance of BE-ABC algorithm in this UCAV path planning scheme, in comparison with two recent state-of-the-art modifications of ABC.

The remainder of this paper is organized as follows. In Section 2, the mathematical model of the combat field is given. In Section 3, the principles of four versions of ABC are introduced in detail. Simulations and the corresponding results are shown in Section 4, together with some remarks. Conclusions are drawn in the last section.

## 2. Combat Field Modeling for UCAV Path Planning

**Figure 1:**
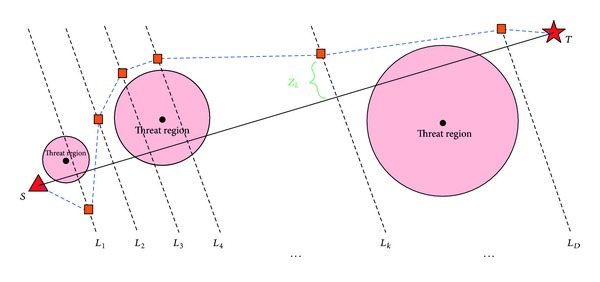
Schematic diagram of combat field modeling.

There are some threatening installations in the combat field, for instance, missiles, radars, and antiaircraft artilleries. The effects of such installations are presented by circles in the combat field of different radiuses and threat weights [36]. If part of its path falls in a circle, an UCAV will be vulnerable to the corresponding ground defense installation. To be more precise, the damage probability of an UCAV is proportional to its distance away from the threat center. Additionally, when the flight path is outside a circle, the probability of being attacked is 0. Let us define the starting point as *S* and the terminal point as *T* (see Figure 1). The UCAV flight mission is to calculate an optimal path from *S* to *T*, with all the given threat regions in the combat field and the fuel consumption considered.

To make this problem more concrete, let us draw a segment *ST* connecting the starting and terminal points first. Afterwards, *ST* is divided into (*D* + 1) equal portions by *D* vertical dash lines {*L*_*k*_,  *k* = 1,2,…, *D*} as plotted in Figure 1. These lines are taken as new axes; then as many as *D* points (see the small rectangles in Figure 1) along such axes will be connected in sequence to form a feasible path from *S* to *T*. In this sense, the *D* corresponding coordinates *Z*_*k*_  (*k* = 1,2,…, *D*) are the variables to be optimized so as to acquire an optimal flight path.

To accelerate the processing speed, it is encouraged to take the *ST* direction as the *x* axis [37]. In this way, point movement on the *L*_*k*_ can be described more easily. Therefore, the first step before the computation of the optimal flight path is the transfer of axes. Any point (*α*_*i*_, *β*_*i*_) on the original combat field gets transferred to (*α*_i_*, *β*_i_*) in the new axes as defined in (1), where *θ* denotes the angle between the original *x* axis and the original *ST* direction, as follows:

(1) $$\begin{array}{c} \left[\begin{array}{c} \alpha_{i}^{*}\\ \beta_{i}^{*} \end{array}\right]=\left[\begin{array}{cc} \cos\theta & \sin\theta \\ -\sin\theta & \cos\theta \end{array}\right]\left[\begin{array}{c} \alpha_{i}\\ \beta_{i} \end{array}\right]. \end{array}$$

Regarding the evaluation of one candidate flight path, the threat cost *J*_threat_ and the fuel consumption *J*_fuel_ are taken into consideration [1], as shown in

(2) J=λ·Jfuel+(1−λ)·Jthreat=λ·∫0lengthwthreat dl+(1−λ)·∫0lengthwfuel dl,

where *J* is the weighted sum of flight cost for this flight path, *λ* ∈ [0,1] represents the weighting parameter, *w*_threat_ and *w*_fuel_ are variables related to every instantaneous position on the path, and length denotes the total length of this candidate flight path.

**Figure 2:**
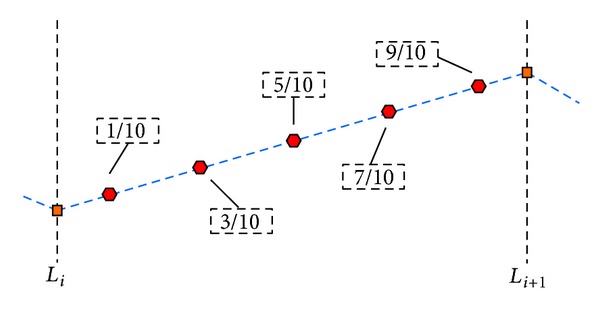
Schematic diagram of flight cost computation.

To simplify the integral operations, the flight cost from the point along *L*_*i*_ to the one along *L*_*i*+1_ is calculated at five sample points [38], as shown in Figure 2.

If the flight path shown above falls into a threat region, the threat cost is calculated as follows:

(3) wthreat,Li→Li+1=lengthi5·∑k=1Nt[tk·(1d0.1,i,k4+1d0.3,i,k4+1d0.5,i,k4     +1d0.7,i,k4+1d0.9,i,k4)],

where *N*_*t*_ denotes the number of threatening circles that the path involves in, length_*i*_ refers to the *i*th subpath length, *d*_0.1,*i*,*k*_^4^ stands for the distance between the 1/10 point on the path and the *k*th threat center, and *t*_*k*_ is regarded as the threat grade of the *k*th threat. In this work, it is assumed that the velocity of the UCAV is a constant. Then the fuel consumption *J*_fuel_ is considered in direct proportion to length (i.e., *w*_fuel_ ≡ 1).

## 3. Principles of ABC Relevant Algorithms

The preceding section holds a description of combat field model. The optimal vector **z** = [*z*_1_, *z*_2_,…, *z*_*D*_] is expected to derive using these intelligent algorithms that will be introduced in detail in this section. The conventional ABC algorithm and three state-of-the-art versions of ABC are applied for the UCAV flight path optimization scheme.

### 3.1. Review of Conventional ABC Algorithm

In ABC, three kinds of bees cooperate to search for the optimal nectar sources in the space, namely, the employed bees, the onlooker bees, and the scout bees [18].

Let **X**_*i*_ = (*X*_*i*_^1^, *X*_*i*_^2^,…, *X*_*i*_^*D*^) represent a candidate position searched by the *i*th employed bee in the space (*i* = 1,…, *SN*). Here, a position refers to a feasible solution for the optimization problem. The number of employed bees is set to *SN*. In each iteration, onlooker bees search locally around those qualified employed bees. The number of onlooker bees is commonly set to *SN*. Here, the qualification standard concerns the roulette selection strategy and will be introduced later in this section. Those employed bees who cannot make any progress for some time will die out and the randomly initialized scout bees will take their places.

At first, all the *SN* employed bees need to be randomly initialized in the feasible solution space. In detail, the *j*th element of the *i*th solution **X**_*i*_ is initialized using

(4) $$\begin{array}{c} X_{i}^{j}⟵X_{\min}^{j}+\text{rand}\left(0,1\right)\cdot \left(X_{\max}^{j}-X_{\min}^{j}\right),\;j=1,2,\ldots ,D, \end{array}$$

where *X*_min⁡_^*j*^ and *X*_max⁡_^*j*^ denote the lower and upper boundaries of this *j*th element and *D* denotes the dimension of any feasible solution.

In each cycle of the iterations, an employed bee executes a crossover and mutation process to share information with one randomly chosen companion and search in the new position X*_i_^*j*^ utilizing

(5) $$\begin{array}{c} {X^{*}}_{i}^{j}⟵X_{i}^{j}+\text{rand}\left(-1,1\right)\cdot \left(X_{k}^{j}-X_{i}^{j}\right). \end{array}$$

In this equation, the *i*th employed bee exchanges information with the *k*th employed bee in the *j*th element. Here, *j* is a randomly selected integer from 1 to *D*. Similarly, *k* is a randomly selected integer from 1 to *SN* while it satisfies the condition that *k* ≠ *i*.

After such crossover and mutation process for the employed bees, the greedy selection strategy will be implemented. If **X**_i_* is better (i.e., its corresponding objective function value is lower), the previous position **X**_*i*_ is discarded; otherwise, the employed bee remains at **X**_*i*_.

Afterwards, an index named *P* is calculated as the qualification reflection for each of the employed bees according to

(6) $$\begin{array}{c} P_{i}=\frac{\text{fitness}\left(i\right)}{\sum_{j=1}^{SN}\text{fitness}\left(j\right)},\\ \text{fitness}\left(i\right)=\{\begin{array}{cc} \frac{1}{1+\text{obj}\left(i\right)} & \text{if}\;\text{obj}\left(i\right)\geq 0\\ 1+\text{abs}\left(\text{obj}\left(i\right)\right) & \text{if}\;\text{obj}\left(i\right)<0. \end{array} \end{array}$$

Here, obj(*i*) denotes the objective function value of the position that the *i*th employed bee currently stays in.

Each of the onlooker bees needs an employed bee to follow. In this case, if *P*_1_ ≥ rand(0,1), the 1st employed bee is chosen for the specific onlooker bee; otherwise, a similar comparison between *P*_2_ and rand(0,1) will be carried on. If all the *P*_*i*_ are smaller than rand(0,1), such process goes over again until one employed bee is selected. In this way, *SN* onlooker bees determine the corresponding employed bees to follow. In this sense, to follow means to search around locally using

(7) $$\begin{array}{c} {Y^{*}}_{0}^{j}⟵X_{0}^{j}+\text{rand}\left(-1,1\right)\cdot \left(X_{k}^{j}-X_{0}^{j}\right). \end{array}$$

In this equation, the *k*th employed bee and the *j*th element are still randomly chosen. Again, the greedy selection strategy is implemented. If the position searched by the onlooker bee is more qualified than the position by the employed bee (i.e., if obj(**Y**_0_*) < obj(**X**_0_)), the employed bee directly moves to the better place.

During the iteration, once an employed bee searches globally but finds no better position, or once an onlooker bee exploits around an employed bee without finding a better position, the invalid trail time *trial* adds one. On the other hand, when any better position can be found for the *i*th employed bee, the corresponding *trial*(*i*) is set to zero instantly. At the end of each iteration, it is necessary to check whether any *trial*(*i*) exceeds a certain threshold named Limit. If *trail*(*i*) > Limit, the *i*th employed bee will be directly replaced by a scout bee. A scout bee simply refers to a randomly initialized position in the food source using (4).

### 3.2. Principle of I-ABC Algorithm

I-ABC algorithm was proposed by Li et al. in 2012 [32]. It differs from the conventional ABC merely in the crossover and mutation equations. Consider

(8) X∗ij⟵Xij·ϕ(i)+(Xkj−Xij)·rand(−1,1) ·ϕ(i)+(globalj−Xij)·rand(0,1),

(9) Y∗0j⟵X0j·ϕ(i)+(Xkj−X0j)·rand(−1,1) ·ϕ(i)+(globalj−X0j)·rand(0,1)·ϕ(i),

(10) $$\begin{array}{c} \phi \left(i\right)=\frac{1}{1+\exp\left({\left(-\text{fitness}(i)/ap\right)}^{\text{iter}}\right)}, \end{array}$$

(11) $$\begin{array}{c} ap\equiv {\text{fitness}(1)|}_{\text{iter}\equiv 1}. \end{array}$$

Equation (8) is designed for global exploration and replaces (5) as in the conventional ABC. Similarly, (9) replaces (7) for the exploitation process. In (10) and (11), global^*j*^ denotes the *j*th element of the best position ever emerged in the cycles of iteration. It is notable that, as in (11), *ap* is randomly determined by the initialization conditions in the first iteration and is usually a relatively small positive number.

### 3.3. Principle of IF-ABC Algorithm

IF-ABC algorithm mainly differs from the conventional ABC algorithm in the utilization of *trial* as the internal feedback information and in the abandonment of the roulette selection strategy [21].

Before the iteration process, as many as *SN* employed bees are randomly sent out to explore in the nectar source space. Particularly, the process to initialize the *j*th element of the *i*th solution **X**_*i*_ is described in (4). Afterwards, the iteration process starts.

In each cycle of iteration, an employed bee executes a crossover and mutation procedure to share information with its one (randomly selected) companion. This procedure is expressed in (5). Then, the greedy selection strategy is implemented so as to select the better position between X*_i_^*j*^ and *X*_*i*_^*j*^ (i.e., to select the one with a relatively higher objective function value). Different from that in the conventional ABC algorithm, the number of elements involved in such crossover and mutation procedure is considered flexible. In other words, (5) should implement on each of the employed bees for *trial*(*i*) times, where *trial*(*i*) ∈ [1, *D*] and will be discussed later. Then the searching procedure by the employed bees in this current cycle is completed, which is usually regarded as the global exploration procedure.

Afterwards, onlooker bees take over the searching process. In the IF-ABC algorithm, each of the employed bees is given a chance to be followed by an onlooker regardless they are “qualified” or not, pursuing to bring about more chances (i.e., more dynamics and diversity) for evolution and to fight against premature convergence. However, qualifications of the employed bees should be taken into account after all. IF-ABC algorithm seeks a new way to evaluate the searching performance.

Now that the roulette selection strategy is discarded in IF-ABC, the onlookers will directly choose their corresponding employed bees to search locally using (12). Here, the companion **X**_*k*_ and the element item *j* (involved in the crossover and mutation procedure) are still randomly selected. Afterwards, the greedy selection strategy is applied on the onlookers to choose between X*_i_^*j*^ and *X*_*i*_^*j*^. Consider

(12) $$\begin{array}{c} {X^{*}}_{i}^{j}⟵X_{i}^{j}+\gamma \left(i\right)\cdot \text{rand}\left(-1,1\right)\cdot \left(X_{k}^{j}-X_{i}^{j}\right), \end{array}$$

where

(13) $$\begin{array}{c} \gamma \left(i\right)=\exp\left\{-\left[trial\left(i\right)-1\right]\cdot \left(\frac{\ln10}{D-1}\right)\right\}. \end{array}$$

For each of the employed bees together with the corresponding onlookers, the parameter *trial* represents the number of inefficient searching times before even one better position is derived. If the *i*th employed bee or the *i*th onlooker bee finds a better position, *trial*(*i*) is directly reset to 1 (but not 0 here); otherwise, it is added by 1. If *trial*(*i*) is larger than *D*, the current *i*th position **X**_*i*_ should be replaced by a reinitialized position using (4).

Since 1 ≤ *trial*(*i*) ≤ *D*, it is expected that as many as *trial*(*i*) elements in a candidate feasible solution will be affected by the exploration process. But when it comes to onlooker bees, only one element is involved because here we believe that multicrossover process contributes little to local search ability [33]. Note that a convergence factor *γ* appears in (12), which is designed to manipulate the exploitation accuracy according to the current convergence status of the *i*th pair of employed bee and onlooker bee. As shown in (13), *γ*(*i*) decreases exponentially to 0.1 as *trial*(*i*) gradually approaches *D*. Here, 0.1 is a user-specified lower boundary of convergence scale, but the selection of such constant can be flexible according to the users. In this sense, the exploitation around one certain employed bee should be gradually intensified before this pair of bees is eventually discarded and replaced by means of reinitialization (when *trial* equals *D*).

To briefly conclude, the variable *trial* in IF-ABC is utilized to manipulate the local exploitation accuracy and to guide the crossover and mutation process in global exploration. Here, convergence performances of the bees are measured not by the corresponding objective function values but by the facts whether they are better than the previous one. In this sense, such change intends for the exploitation of positions where unqualified employed bees stay in.

**Pseudocode 1:**
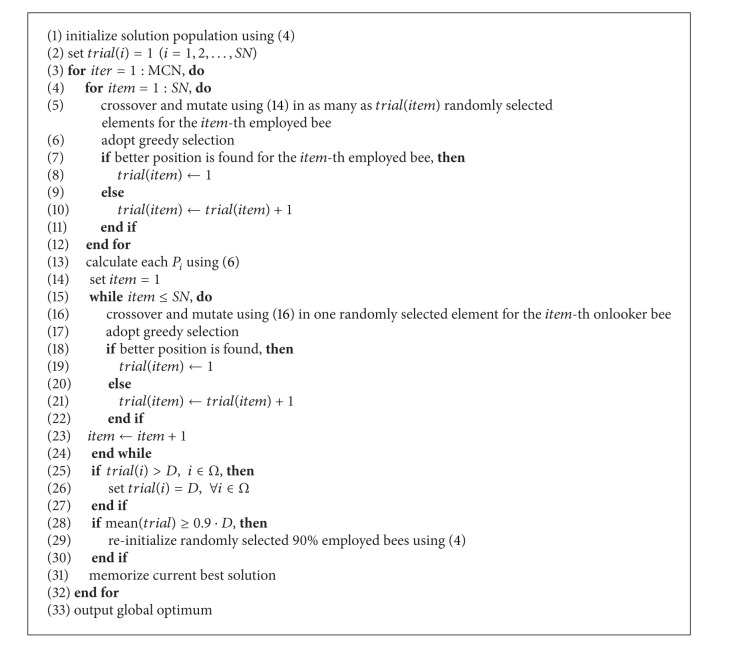


### 3.4. Principle of BE-ABC Algorithm

BE-ABC algorithm mainly differs from IF-ABC algorithm in two aspects. One is the utilization of the parameter *trial*(*i*) to manipulate the exploration/exploitation accuracy and the other is a new strategy for the generation of scout bees [39].

Regarding the exploration procedure, a convergence factor is added in the crossover and mutation equation to manipulate the exploration accuracy. Besides, the number of elements involved in the crossover and mutation process is designed to be adaptable.

In detail, the *j*th element of the *i*th employed bee changes to be X*_i_^*j*^ as defined in the following:

(14) $$\begin{array}{c} {X^{*}}_{i}^{j}⟵X_{m}^{j}+\text{rand}\left(0,1\right)\cdot \left(X_{k}^{j}-X_{i}^{j}\right)\cdot \mu \left(i\right), \end{array}$$

(15) $$\begin{array}{c} \mu \left(i\right)=\frac{trial\left(i\right)}{trial\left(i\right)+trial\left(k\right)}, \end{array}$$

where *j* ∈ [1, *D*], for all *m*, *k* ∈ [1, *SN*], *trial*(*i*) ∈ [1, *D*], and *i* ≠ *k*.

*i* is not necessarily equal to *k* as shown in (14). Here, such modification intends to provide more dynamics for the global exploration. In addition, it is also notable that the lower boundary of *trial*(*i*) is 1 (like that in IF-ABC). Then *μ*(*i*) ∈ [1/(*D* + 1), *D*/(*D* + 1)]⊆(0,1) is regarded as a manipulator for the exploration, which reflects the convergence efficiency of the *i*th employed bee. Besides, the upper boundary of each *trial*(*i*) is set to *D*; then it is required that as many as *trial*(*i*) out of the *D* elements will be involved in such crossover and mutation procedure for the *i*th employed bee. Such idea may be intuitively interpreted as follows: a relatively large *trial*(*i*) corresponds to a relatively inefficient *i*th employed bee. Therefore, by changing more elements at one time, the *i*th employed bee gradually becomes distrustful of its current position. It is similar regarding the definition of *μ*(*i*). If *trial*(*i*) is small, *μ*(*i*) will be relatively small (when *trial*(*k*) is temporarily fixed). In this sense, the exploration process is more likely to be an exploitation process. In this work, it is believed that there should not be an explicit difference between the exploration and exploitation procedures. In other words, when the exploration/exploitation ability needs to be enhanced, the searching system should be capable of adaptively catering for such demands. In this sense, the proposed BE-ABC algorithm aims to effectively capture a balance between the exploration and the exploitation, so as to make the evolution efficient.

After such crossover and mutation process for the employed bees, the greedy selection strategy will be implemented. If **X**_i_* (defined in (14)) is more qualified, the previous position **X**_*i*_ is discarded and *trial*(*i*) is set to 1; otherwise, the employed bee remains at **X**_*i*_.

Regarding the onlooker bees, only one element in the solutions will be changed at one time using (16) so as to guarantee that such procedure is sufficiently “local” as follows:

(16) $$\begin{array}{c} {Y^{*}}_{0}^{j}⟵X_{0}^{j}+\text{rand}\left(-1,1\right)\cdot \left(X_{k}^{j}-X_{0}^{j}\right)\cdot \mu \left(i\right). \end{array}$$

Similarly, it is assumed that the local exploitation needs to be intensified (by using *μ*(*i*)) when *trial*(*i*) is large. Afterwards, the greedy selection is implemented. If **Y**_i_* is more qualified, the previous position **X**_*i*_ is discarded and *trial*(*i*) is set to 1; otherwise, *trial*(*i*) adds one.

In each cycle of iteration, after the exploration and the exploitation procedures, any *trial*(*i*) that exceeds *D* will be reset to *D* (rather than generating scout bees in the conventional ABC). Before it proceeds to the next cycle, the average value of *trial* (i.e., (1/*SN*)∑_*i*=1_^*SN*^ *trial*(*i*)) is compared with 90% · *D*. If 90% · *D* is smaller, which indicates that the overall iteration system is not functioning efficiently, the overall degradation procedure will be carried out. If not, it directly proceeds to the next cycle of iteration. Here, 90% is a user-specified parameter to determine the degree of the inefficient evolution.

In detail, 90% · *SN* randomly selected employed bees will be reinitialized using (4) in such overall degradation procedure. At the same time, the corresponding *trial*(·) will be reset to 1 as well. This idea is named overall degradation strategy in contrast with the conventional rule for scout bees in ABC.

The pseudocode of BE-ABC for numerical optimization is given in Pseudocode 1. 

## 4. Experimental Results and Discussions

In order to investigate the efficiency and robustness of these ABC relevant algorithms, three simulation cases have been investigated. All the contrast experiments involved in this section were implemented with MATLAB R2010a and each kind of experiment was repeated 50 times with different random seeds. It is constantly set that *λ* = 0.5, *SN* = 30, and Limit = 30.

**Figure 3:**
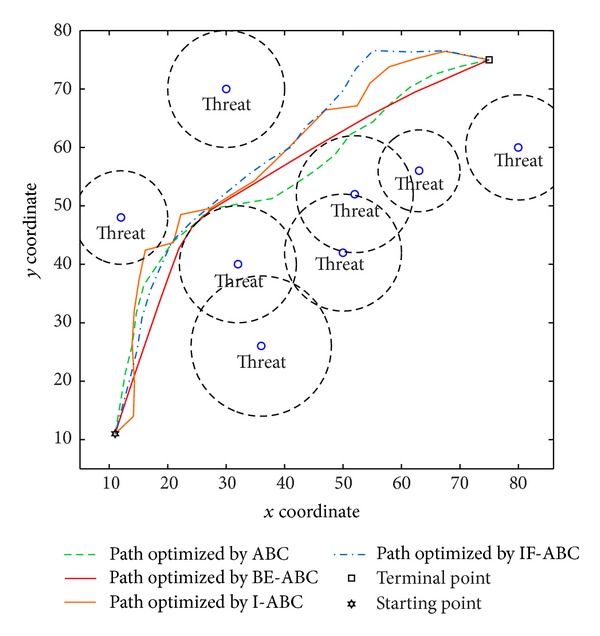
Comparative path planning results optimized by different ABC relevant algorithms in Case 1 (*D* = 20).

**Figure 4:**
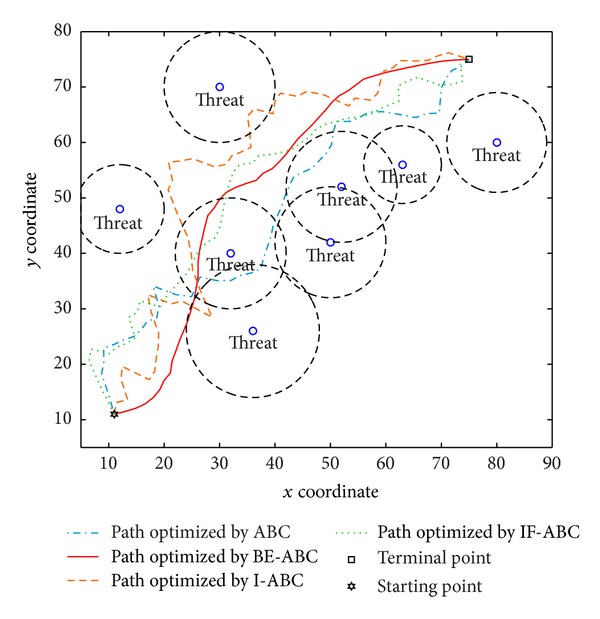
Comparative path planning results optimized by different ABC relevant algorithms in Case 1 (*D* = 50).

**Figure 5:**
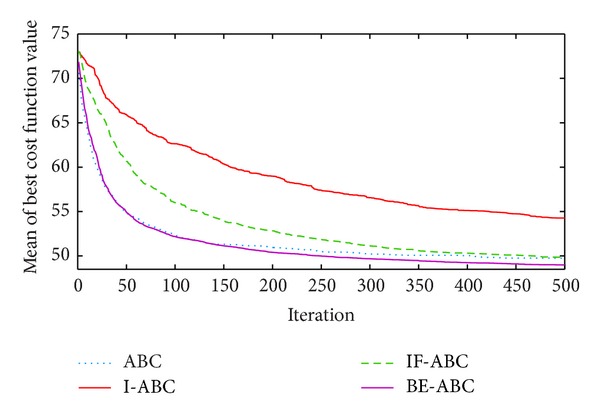
Comparative convergence curves of ABC, I-ABC, IF-ABC, and BE-ABC in Case 1 (*D* = 20 and MCN = 500).

**Figure 6:**
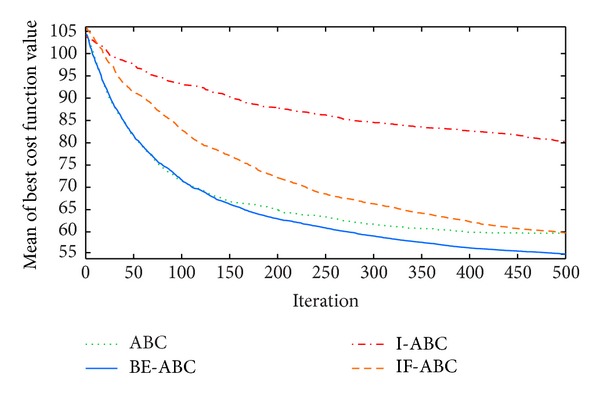
Comparative convergence curves of ABC, I-ABC, IF-ABC, and BE-ABC in Case 1 (*D* = 50 and MCN = 500).

**Figure 7:**
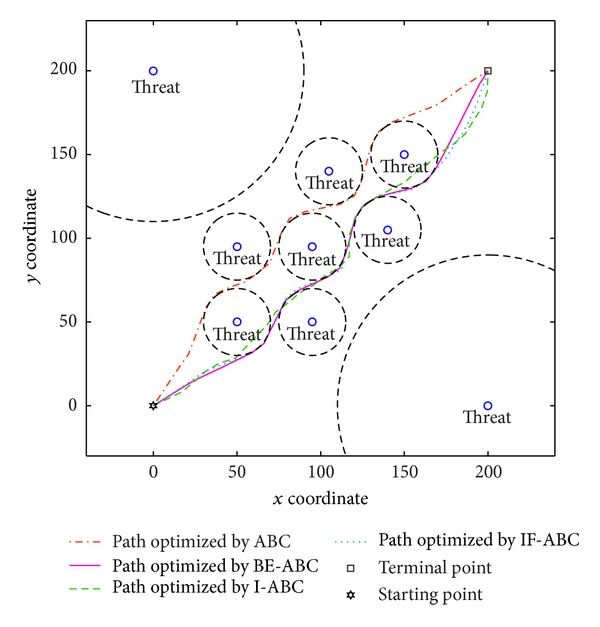
Comparative path planning results optimized by different ABC relevant algorithms in Case 2 (*D* = 30 and MCN = 1000).

**Figure 8:**
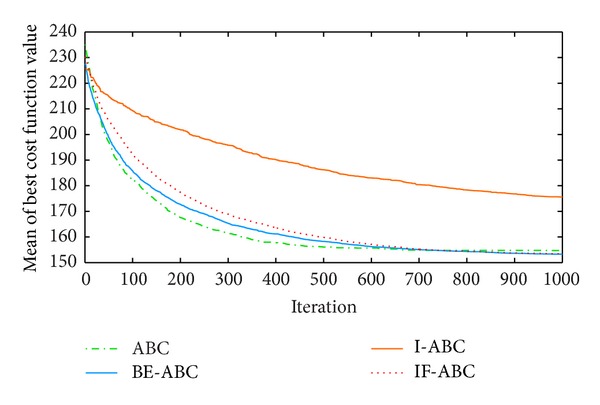
Comparative convergence curves of ABC, I-ABC, IF-ABC, and BE-ABC in Case 2 (*D* = 30 and MCN = 1000).

**Figure 9:**
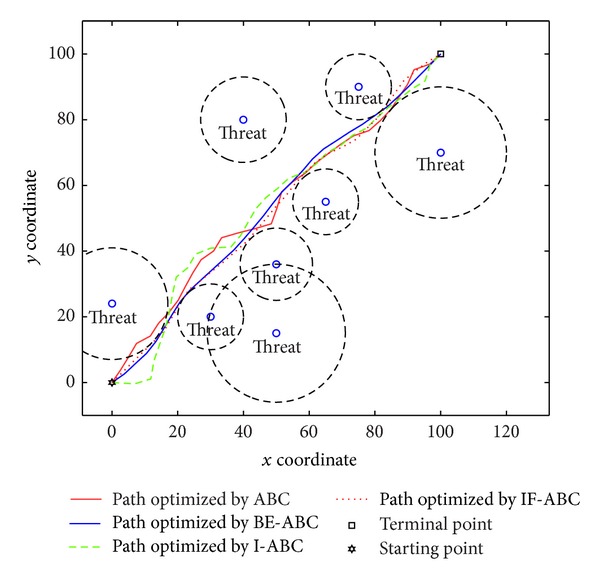
Comparative path planning results optimized by different ABC relevant algorithms in Case 3 (*D* = 30 and MCN = 1000).

**Figure 10:**
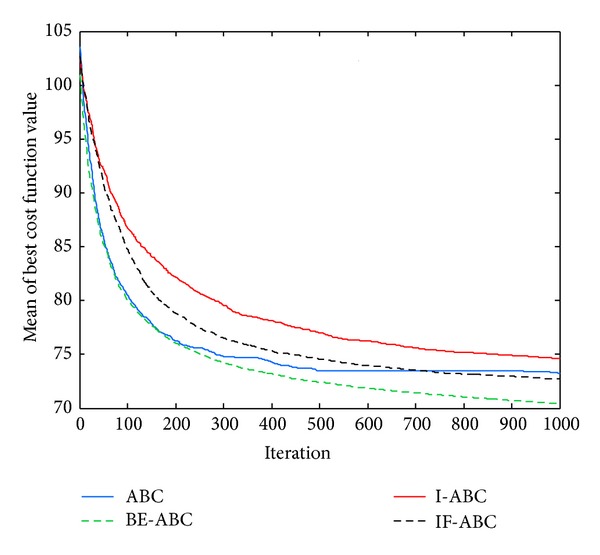
Comparative convergence curves of ABC, I-ABC, IF-ABC, and BE-ABC in Case 3 (*D* = 30 and MCN = 1000).

In the first case, the starting point is set to (11,11) and the terminal point is set to (75,75) [12]. In the second and third cases, the starting points are both (0,0) and the terminal points are (200,200) and (100,100), respectively. The threat locations and threat grades for the three cases are listed in Table 1. Some typical simulation results are demonstrated in Figures 3, 4, 5, 6, 7, 8, 9, and 10. In detail, Figures 3 and 4 show the comparative simulation results when *D* = 20 and *D* = 50, respectively, for Case 1. The corresponding convergence curves are shown in Figures 5 and 6. Similarly, paths optimized by different algorithms in Cases 2 and 3 are shown in Figures 7 and 9, respectively, when *D* = 30. Their corresponding convergence curves are plotted in Figures 8 and 10. Complete evaluations of convergence performances (i.e., the mean and the standard deviation of the threat cost function values) are listed in Table 2.

Regarding the paths plotted in Figures 3, 4, 7, and 9, the trajectories optimized by BE-ABC are usually more smooth and advantageous. Specifically, as shown in Figure 7, the path optimized by the conventional ABC happened to be a local optimal solution.

Viewing the results in Table 2 and the comparative curves in Figures 5, 6, 8, and 10, we may notice that the superiority of BE-ABC gradually show up when the dimension *D* increases. The situation is similar but not so significant regarding IF-ABC. It is because the roulette selection strategy is discarded in IF-ABC, abandoning the feedback information hiding in the objective function values. In a way, IF-ABC sacrifices part of its ability to converge fast for the competence to converge globally [33]. In contrast, improvements made in BE-ABC are relatively moderate and mild, which may account for its good convergence performance.

In the meantime, in some early cycles of iteration, the convergence speed of BE-ABC tends to be roughly the same with that of the conventional ABC. Such phenomenon may be due to the fact that, during the early cycles of iteration, it is relatively easy to evolve for each of the employed bees; that is, *trial*(*i*) are not large in general. Therefore, BE-ABC is similar with the conventional ABC in the convergence performance. However, as the iteration process continues, the efficiency of ABC will be impacted by the obstacles of local optimums. At the same time, the modifications in BE-ABC algorithm make sense.

## 5. Conclusion

In this paper, BE-ABC algorithm is applied for the UCAV path planning optimization problem. Simulation results clearly indicate that BE-ABC shows more stability and efficiency in this two-dimensional flight path planning optimization scheme than ABC, I-ABC, and IF-ABC.

BE-ABC algorithm intends to fully utilize the convergence status within the iteration system so as to manipulate the searching accuracy and also to strike a balance between the local exploitation with global exploration. Previous studies concerning the improvements of ABC always focused on the remedies from the outside world, neglecting the true convergence status hiding in the internal iteration process. In this sense, this work can be regarded as publicity for such idea. Our future work will cover some further comparisons with more state-of-the-art intelligent algorithms.

## Figures and Tables

**Table 1:** Information of threat installations in combat field.

Case number	Threat center location	Threat radius	Threat grade
1	[52, 52]	10	2
	[32, 40]	10	10
	[12, 48]	8	1
	[36, 26]	12	2
	[80, 60]	9	3
	[63, 56]	7	5
	[50, 42]	10	2
	[30, 70]	10	4

2	[0, 200]	90	7
	[200, 0]	90	7
	[50, 50]	20	5
	[95, 95]	20	5
	[150, 150]	20	5
	[95, 50]	20	6
	[50, 95]	20	5
	[140, 105]	20	6
	[105, 140]	20	5

3	[30, 20]	10	5
	[50, 15]	21	5
	[65, 55]	10	5
	[0, 24]	17	5
	[50, 80]	27	5
	[75, 90]	10	5
	[100, 70]	20	5
	[50, 36]	11	5

**Table 2:** The mean and standard deviations of cost function values.

Case number	MCN	*D*	ABC		I-ABC		IF-ABC		BE-ABC	
			Mean	S.D.	Mean	S.D.	Mean	S.D.	Mean	S.D.
1	500	20	49.8813	0.5976	54.3889	3.6656	49.7975	0.6393	**48.9558**	**0.5957**
	500	30	52.9887	1.4259	60.4591	3.2883	51.9396	1.3434	**50.4567**	**0.6726**
	500	50	59.9722	2.9133	80.1747	7.7575	59.9569	**2.2149**	**54.9826**	2.2310

2	1000	20	161.7459	2.5523	171.6983	2.1826	160.1839	**0.9241**	**160.0276**	1.5195
	1000	30	154.9276	2.5123	185.7927	3.7547	153.5837	**0.4925**	**153.4103**	0.5549
	1000	50	157.2044	3.6031	164.9908	2.8118	157.8147	1.9058	**153.5245**	**1.0479**

3	1000	20	73.5090	0.2486	73.7209	0.4729	73.0254	0.1079	**72.8889**	**0.1403**
	1000	30	73.9346	1.0134	74.8452	0.8904	73.6928	**0.4083**	**70.0789**	0.4634
	1000	50	78.8720	3.1422	81.8885	2.7718	77.4828	2.0989	**75.8906**	**1.3950**

Those bold values denote the best solutions (mean or S.D.) among four algorithms in every single case.
